# Delivery motorbike crash injuries in South Africa: A scoping review and call for policy reform

**DOI:** 10.4102/jphia.v17i1.1822

**Published:** 2026-08-06

**Authors:** Pududu A. Rachuene, Falethu Sukati, Mthunzi Ngcelwane

**Affiliations:** 1 Department of Orthopaedics, School of Medicine, Faculty of Health Sciences, Steve Biko Academic Hospital, University of Pretoria, Pretoria, South Africa

**Keywords:** motorbike delivery drivers, road traffic crashes, musculoskeletal injuries, orthopaedic trauma, public health burden, economic burden, South Africa

## Abstract

**Background:**

Road crashes continue to cause morbidity and mortality in South Africa, with motorbike accidents causing serious musculoskeletal injuries. The rapid growth of e-hailing delivery services has increased the number of motorbike drivers on congested urban roads, increasing their vulnerability to crashes.

**Aim:**

This scoping review examined South African motorbike delivery driver road traffic crashes, injury patterns, economic implications, and related regulatory gaps.

**Setting:**

South African public healthcare and trauma systems.

**Methods:**

A systematic search of PubMed, Scopus, and Web of Science was performed for studies published between 2020 and 2025. The search strategy combined Medical Subject Headings and Emtree terms pertaining to motorbikes, delivery services, and injury-related outcomes. Eligible English-language studies were included, with data extracted using a standardised collection sheet and synthesised descriptively.

**Result:**

The review comprised three studies with 133 participants with 161 orthopaedic injuries. The studies conducted in Cape Town, Tembisa, and Pretoria consistently reported that delivery motorbike crashes result in high-energy trauma predominantly affecting young, male riders. The predominant injuries consisted of severe long-bone fractures and polytrauma in patients. Across sites, most patients required surgical intervention, and intensive care unit (ICU) admissions were necessary in the most severe cases. The treatment of these patients incurred substantial costs. Expenses were predominantly influenced by surgical procedures, prolonged hospitalisation, ICU treatment, and implants.

**Conclusion:**

Motorbike delivery drivers in South Africa face disproportionate health risks and financial insecurity because of traffic-related injuries.

**Contribution:**

This review highlights important policy and legislative gaps and supports reforms aimed at improving occupational protection and road safety for delivery drivers.

## Introduction

Road traffic crashes pose a substantial public health and economic challenge in South Africa. In addition to the immediate human cost of deaths and major injuries, these events put a lot of stress on the healthcare system, which often needs rapid trauma care and long-term rehabilitation services. Road crashes cause damage to infrastructure and vehicles, loss of production because of injuries or deaths, and financial problems for individuals and communities.^1,2,3^ As a result, billions of rand are lost annually nationwide because of medical costs, insurance claims, litigation, productivity losses, and public expenditure related to emergency response and damaged infrastructure restoration. The overall effect harms both social and economic growth, underscoring the importance of using evidence-based methods to reduce the number and severity of road traffic accidents. During the coronavirus disease of 2019 (COVID-19) pandemic, there was a temporary decrease in both trauma-related hospital admissions and accidents because of restrictions on traffic volume and movement.^4^ This decrease illustrated the potential of policy and regulatory interventions to significantly mitigate road-related injuries.

Nevertheless, the rapid proliferation of e-hailing delivery services for food and supplies has introduced new obstacles since 2020.^5^ These motorcyclists are often forced to navigate already congested urban roads, particularly during peak traffic hours, placing them at risk of crashes and injuries. This trend raises significant concerns about the socioeconomic security of motorbike delivery couriers and road safety. Numerous individuals are employed under precarious conditions, frequently classified as independent contractors rather than employees. This classification restricts their access to occupational protections, healthcare benefits, and compensation in the event of an injury.^6^ These dynamics underscore the need for a comprehensive analysis of the available evidence.

This scoping review seeks to synthesise the existing literature on motorbike-related delivery accidents and musculoskeletal injuries in South Africa, with the dual objectives of mapping injury patterns among these patients and assessing the associated clinical and economic costs, while advocating for the recognition of delivery drivers as employees and the necessity for health insurance. By mapping the evidence, this review seeks to contribute to policy discussions on the protection of vulnerable workers in the growing e-hailing services and economy.

## Methods

### Search strategy and eligibility criteria

A comprehensive scoping review of the literature was conducted using databases – PubMed (NCBI, Bethesda, MD, United States [US]), Scopus (Amsterdam, the Netherlands), and Web of Science (London, United Kingdom [UK]). Eligible studies were limited to those conducted in South Africa or involving South African populations and published or available in English translation between 2020 and 2025. The reference lists of included articles were examined to identify any other pertinent articles. The search strategy was designed with a combination of Medical Subject Headings and Emtree terms. Search criteria included topics pertaining to motorbike, motorcycle, or scooter utilisation; delivery or food-delivery services; and outcomes such as injury, trauma, fracture, or musculoskeletal injury (Table 1). All records obtained were evaluated in accordance with the Preferred Reporting Items for Systematic Reviews and Meta-Analyses guidelines.

**TABLE 1:** A summary of the key characteristics, design, setting, and aims of the three identified studies focusing on orthopaedic trauma sustained by delivery motorcyclists in South Africa.

Characteristic	Cape Town study^10^	Tembisa study^8^	Pretoria study^9^
Study design	Prospective case series	Prospective cross-sectional study	Retrospective qualitative observational study
Level of evidence	Level 4	Level 3	Level 4
Setting	Cape Town metropole (New Somerset, Victoria, Groote Schuur, and Mitchells Plain District hospitals)	Tembisa Provincial Tertiary Hospital (TPTH), Ekurhuleni Municipality	Steve Biko Academic Hospital (SBAH) in Pretoria, Tshwane Municipality
Study period	12 months, between June 2022 and June 2023	7 months, from 01 July 2022 to 31 January 2023	3 years, between 01 January 2020, and 31 December, 2022
Total drivers studied (*n*)	44	60	29
Total injuries reported (*n*)	57 injuries	65 injuries	39 injuries
Primary aim	To estimate the costs to the state healthcare system of the initial orthopaedic surgical management of uninsured motorcyclists.	To investigate the cumulative incidence of orthopaedic-related trauma in e-hailing motorcycle drivers.	To determine the pattern and severity of orthopaedic injuries due to motorbike delivery accidents
Funding and conflicts	No funding received; no conflicts of interest declared.	No funding received; no conflicts of interest declared.	No funding received; no conflicts of interest declared.

Note: Please see the full reference list of the article Rachuene PA, Sukati F, Ngcelwane M. Delivery motorbike crash injuries in South Africa: A scoping review and call for policy reform. J Public Health Africa. 2026;17(1), a1822. https://doi.org/10.4102/jphia.v17i1.1822, for more information.

### Screening process

Two reviewers independently examined titles and abstracts utilising the Rayyan QCRI tool (Cambridge, MA, US).^7^ Articles were included only after consensus among reviewers was reached.

### Data extraction

Data extraction was conducted with a standardised collection sheet. References were compiled using Mendeley v1.19.8 (London, UK). Each reviewer performed data extraction independently, and any inconsistencies were reconciled through discussion until consensus was reached. Selected full-text publications were meticulously evaluated, and the retrieved data were compiled into a Microsoft Excel spreadsheet (Redmond, WA, US) for analysis.

## Review finding

### Selection of studies

**FIGURE 1:**
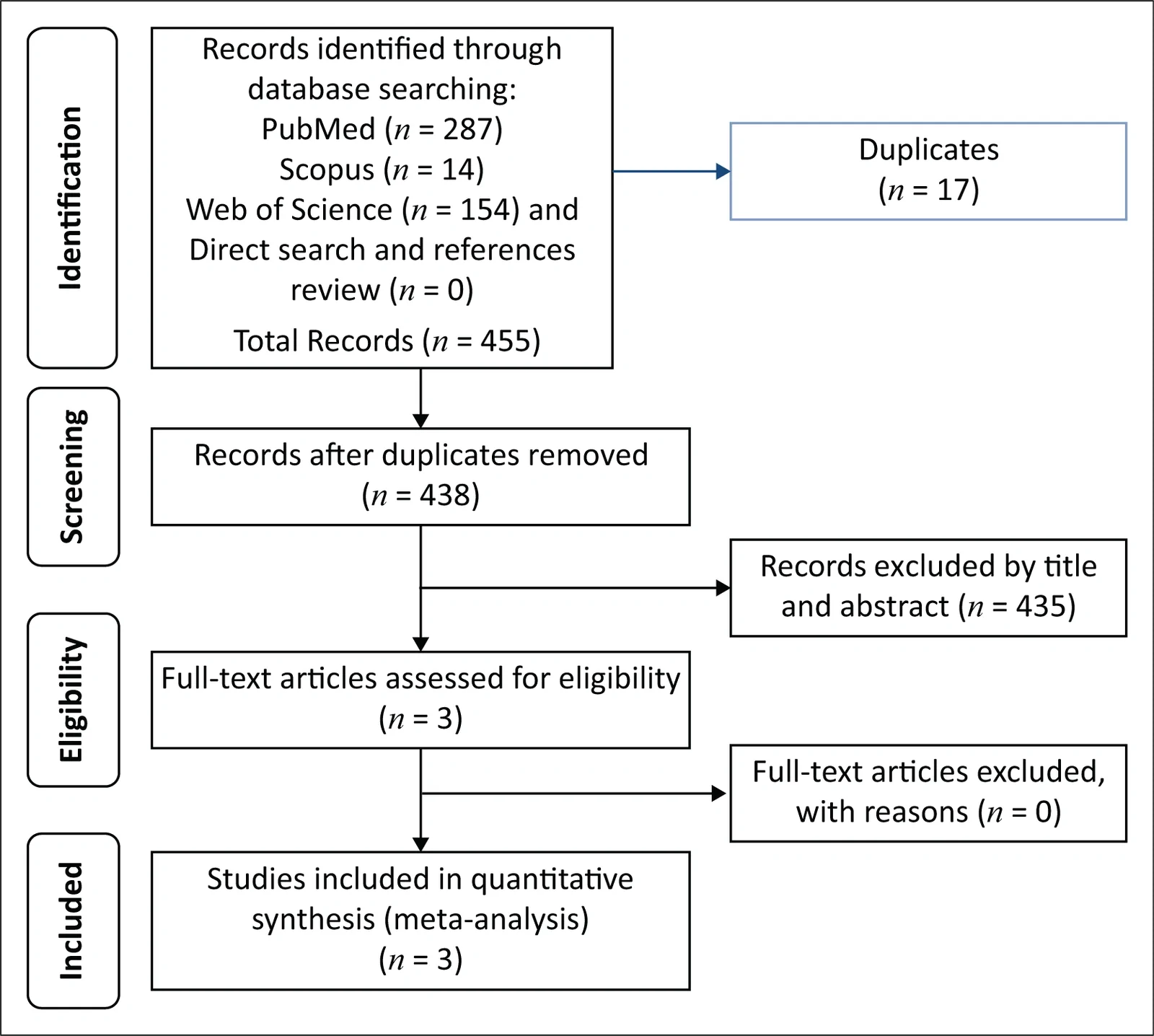
Flow chart of the included studies in this review.

Our search strategy revealed 287 studies from PubMed, 14 from Scopus, and 154 from Web of Science. Following the elimination of duplicates, a total of 438 records were retained for screening. A total of 435 studies failed to satisfy the inclusion criteria and were therefore excluded after the comprehensive screening process. Three studies satisfied the inclusion criteria (Figure 1) and their characteristics are presented in Table 1.

### Study characteristics

Three studies,^8,9,10^ reporting on 161 injuries in 133 patients, were included in this review. The distribution and severity of orthopaedic injuries sustained by delivery motorbike drivers were analysed across three distinct studies in South African public hospitals: a study focusing on Cape Town metropole, a study conducted at Tembisa Provincial Tertiary Hospital (TPTH) focusing on Ekurhuleni municipality, and a study conducted at Steve Biko Academic Hospital in Tshwane municipality. All studies assessed injury patterns in victims of motorbike accidents, whereas two studies examined the associated economic effects. The main features of these investigations are presented in Table 1.

### Clinical burden on the orthopaedic services

The injuries sustained by delivery motorcyclists are characterised by high-energy mechanisms, severe orthopaedic injuries, and a significant demand on hospital resources. The cumulative incidence of injuries sustained by this group was reported as 118 per 10 000 patients (1% of total orthopaedic trauma) treated in Tembisa over 7 months (Pretoria and Cape Town). This population group consists almost exclusively of young, healthy males (mean age 32 years in one study; 20–44 years, with an average age of 29 years, in another). In the Cape Town study, all included patients were foreign nationals. The vast majority are considered self-employed (86% in the Cape Town study) via mobile phone applications, such as Uber, Mr Delivery, Checkers Sixty60, and Bolt. These findings are summarised in Table 2.

**TABLE 2:** A summary of the clinical characteristics and injury patterns observed in victims of delivery motorbike crashes.

Location	Cape Town study^10^	Tembisa study^8^	Pretoria study^9^
Study population	Uninsured delivery motorcyclists requiring orthopaedic surgical intervention	E-hailing motorcycle drivers presenting with orthopaedic trauma (surgical and conservative)	Delivery motorbike riders presenting with orthopaedic injuries
Proportion sustaining bony injuries (fractures/dislocations)	Predominantly fractures of the femur and tibia	47 drivers experienced fractures or dislocations	77% of injuries (30/39) were bony injuries
Specific major fractures (as % of total injuries)	Femur fractures: 36% (21 of 57 injuries)	Reported as a percentage of lower limb injuries	Femur fractures: 37.9% (of 39 total injuries); 52% of lower limb injuries
	Tibia fractures: 24% (14 closed, 8 open) (14 of 57 injuries)	Reported as a percentage of lower limb injuries	Tibial fractures: 9% of lower limb injuries; 17.35% of long bone fractures
Open fractures	9 of 57 injuries (6%) were open fractures (8 tibia, 1 femur)	Not specifically quantified as a percentage of total injuries	20% of all fractures (6 out of 30 fractures)
Upper limb injuries	18% of total injuries	28 injuries	7 injuries
Polytrauma	29% classified as polytrauma (ISS > 15)	Injuries suggestive of ‘more high velocity in nature’	32% sustained multiple injuries; 6% admitted to ICU/HCU
Required surgical management	100% (cohort defined by need for surgery)	34 injuries (30 drivers) required surgical management and admission	86% of patients (25 out of 29) were managed surgically
ICU admission rate	13% of patients required ICU admission	Not quantified in total patients	3.45% (1 patient to ICU, 1 HCU)

Note: Please see the full reference list of the article Rachuene PA, Sukati F, Ngcelwane M. Delivery motorbike crash injuries in South Africa: A scoping review and call for policy reform. J Public Health Africa. 2026;17(1), a1822. https://doi.org/10.4102/jphia.v17i1.1822, for more information. Percentages for the TPTH regarding specific limb fractures (e.g. 32% tibia fracture) refer to the distribution within the lower limb injury category, not the total injuries sustained by the cohort. The SBAH study percentages are based on both the total number of injuries (*n* = 39) and the subset of lower limb injuries.

HCU, high care unit; ICU, intensive care unit; ISS, injury severity score; TPTH, Tembisa Provincial Tertiary Hospital; SBAH, Steve Biko Academic Hospital.

Accidents are common after hours during peak traffic, with 60% of patients in one study arriving at the emergency department after 16:00. The predominant mechanism of injury reported was the motorbike being struck by another vehicle. The majority (77% in Pretoria, 78% in Tembisa) included long bone fractures and/or major joint dislocations. The injuries are often high velocity in nature and include femur fractures (36% of 57 injuries in Cape Town study; 52% of lower limb injuries in Pretoria study, 26% in Tembisa) and tibial fractures (32% in Tembisa study, 24% in Cape Town study). Open fractures are reported in all studies, including those involving the tibia and femur.

For this review, polytrauma was defined as an Injury Severity Score (ISS) greater than 15 or, when ISS was unavailable, as the presence of injuries affecting multiple body systems requiring admission to intensive or high-level care. Polytraumatised patients constituted 29% of the injured drivers in the Cape Town study, with 13% requiring admission to an intensive or high care unit. An overwhelming majority of these patients required surgical management. In one study, 86% of delivery motorcyclists were managed surgically. The most common procedure was long bone intramedullary nailing. The majority had a prolonged hospital stay: the median length of admission was 8 days in the Cape Town study, and over 50% of patients in the Pretoria study stayed longer than 5 days. Prolonged hospitalisation increases the consumption of hospital resources and affects patients’ productivity.

### Economic burden

The initial orthopaedic surgical management of 44 uninsured delivery motorcyclists in Cape Town over 1 year cost the state an estimated R3.99 million. The median cost per injured driver for initial surgical management was R52 061.00 (~United Stated dollar [USD] 3183.00) (with a range up to R348 894.00 [~USD21 347.00]). In Gauteng, an estimated total treatment cost of R2 781 941.70 (~USD170 217.00) was incurred for 30 surgically managed e-hailing drivers at TPTH over 7 months. This contributed 0.0047% of the provincial health budget for 2022/2023. These costs are resource-intensive, driven by factors, such as prolonged hospital and intensive care unit (ICU) stays. Total costs for in-hospital admission and ICU stay in the Cape Town study amounted to over R2m (~USD122 373.00) (median length of ICU stay was 4 days, and median cost of R80 045.00) ~ (USD4897.00). The total cost of implants was R1.33m (~USD81 000.00), and the cost of imaging, including X-rays, computed tomography scans, and fluoroscopy, was estimated at R417 594.00 (~USD25 551.00).

### Quality assessment

Consistent with standard scoping review methodology, a formal risk-of-bias assessment was not undertaken.^11^ The aim of this review was purely to map the existing literature and identify knowledge gaps, rather than to evaluate the effectiveness of interventions.

## Implications and recommendations

We recognise the limitations of this scoping review and those inherent in the included studies. The studies are geographically limited to Gauteng and Cape Town, concentrate solely on orthopaedic injuries within the public healthcare system, and omit non-surgical cases and pre-hospital fatalities, resulting in inadequate generalisability and under-representation of the actual burden. The available evidence is geographically limited to specific municipalities in Gauteng (Ekurhuleni and Tshwane) and the Western Cape (the Cape Town metropolitan area). No studies were identified from other provinces, such as KwaZulu-Natal or the Eastern Cape. Methodological limitations, such as inadequate and retrospective data, and a lack of long-term studies, undermine the reliability of the data. Financial data are inconsistent and systematically undervalued, with essential cost elements such as emergency care, follow-up, and related trauma treatments omitted, and cost assessments derived from obsolete or secondary sources. The studies were also not assessed for methodological quality and levels of bias. However, this review outlines general injury patterns and highlights the health and economic burden associated with these crashes, and identifies gaps in the literature to guide future research on this subject.

### Discussion

This review draws on recent South African literature examining orthopaedic injuries sustained by delivery motorbike drivers, summarising the observed clinical and economic burdens, and detailing subsequent policy and legislative advocacy. Since the COVID-19 pandemic, there has been a reported increase in the number of these drivers on South African roads. The job incentivises drivers to work long hours, often in adverse weather and low-visibility conditions, further increasing their risk of crashes.

The injury patterns observed in this review are consistent with high-energy mechanisms typically seen in road traffic trauma in low- and middle-income countries (LMICs). Long-bone fractures of the femur and tibia were among the most frequently reported injuries across the included studies, reflecting the vulnerability of motorbike riders during collisions with larger vehicles. These injuries frequently require operative fixation and prolonged hospitalisation, placing substantial strain on already resource-limited orthopaedic trauma services. Similar patterns have been reported in other trauma registries in LMICs, where road traffic injuries disproportionately consume trauma theatre capacity and intensive care resources. Strengthening trauma systems, including improved dedicated orthopaedic trauma capacity, could reduce morbidity and disability associated with these injuries. Evidence from LMIC trauma system strengthening initiatives has demonstrated that improved trauma care infrastructure can significantly reduce mortality following road traffic injuries.^12^

The impacts of these crashes extend beyond the direct hospital costs. The orthopaedic injuries sustained place a significant financial burden on the public healthcare sector, particularly because most injured drivers are uninsured. The reported costs are likely to be underestimates of the true burden, as they exclude costs related to the emergency centre, treatment of associated non-orthopaedic systemic injuries (such as head injuries), outpatient care, chronic care, follow-up, re-admissions, and general consumables. The resources consumed by managing these severe trauma cases divert theatre time and capacity away from other hospital services, such as elective surgery. Because many drivers lack formal employment contracts, the costs that would typically be claimed from an occupational injury compensation fund are instead borne by the state medical system.

They also have a direct economic effect because of the loss of productivity among a young, economically active demographic. The injured drivers are often breadwinners, and their inability to earn an income for weeks or months post-injury creates a societal problem.

In South Africa, e-hailing drivers are generally considered independent contractors. They are therefore typically not entitled to the full range of employee benefits, including those related to occupational health and safety.^6^ The *National Land Transport Amendment Act, 2023 (Act No. 23 of 2023, assented to in June 2024)* (NLTAA) and *the Occupational Health and Safety Act, 1993 (OHSA) (Act No. 85 of 1993)*, intersect with the reality of e-hailing drivers primarily through regulation, vehicle safety requirements, and the contentious issue of worker classification and protection.^13^ The NLTAA specifically inserts the definition of an ‘electronic hailing service’ or ‘e-hailing service’ – regulation of the E-hailing Application (Section 66A).^14^ This section, however, has no specific provisions or descriptions for food-delivery e-hailing drivers. It provides for a simplified procedure for obtaining temporary licences ‘over the counter’ and a simplified application process for replacing a vehicle specified in the operating licence. A person or an organisation that provides the e-hailing software application (apps) commits an offence if they permit an operator to use the app to operate a vehicle without a valid operating licence or permit or if it has lapsed or been cancelled. We suggest that specific policy reforms should include amendments to the NLTAA to incorporate provisions for delivery motorbike operators and the extension of occupational protection under the *Compensation for Occupational Injuries and Diseases Act* (COIDA) to delivery motorbike drivers.

If e-hailing drivers were recognised as employees, they would be entitled to OHSA protections. They would also be entitled to education on labour laws, safety equipment and maintenance, as well as training on the hazards associated with their work, among other benefits. Many e-hailing drivers are young migrant workers who frequently lack formal safety training and work under poorly regulated conditions. These factors may predispose them to unsafe practices, increase their risk of road traffic crashes, and leave them with limited occupational risk recognition, injury insurance, and unemployment protection.

The clinical and economic burdens identified in the literature lead to specific recommendations for policy and legislative change, primarily focusing on mandated insurance and on provisions for benefits and protections for self-employed delivery drivers similar to those afforded to employees. The core highlighted issue is that many delivery drivers are considered self-employed through apps and lack formal employment contracts, leaving them without recourse to claim under the COIDA in the event of an accident. We strongly advocate amending existing legislation and policies to protect this vulnerable demographic and shift the financial burden away from the state. Legislative reforms mandating insurance coverage for delivery motorbike drivers should be considered. The medical expenses incurred by the public sector would otherwise have been claimed from the occupational injury compensation fund had the drivers held formal contracts.

## Conclusion

This review highlights the substantial musculoskeletal injuries sustained by motorbike delivery drivers because of road traffic crashes in South Africa, along with the considerable economic burden borne both by affected individuals and the public healthcare system. The findings underscore the urgent need for targeted interventions that address road safety, occupational health, and social protection. We call for policy and legislative reforms that formally recognise delivery drivers as employees, ensuring that they are afforded adequate protection, benefits, and compensation. Strengthening these safeguards is essential not only to protect vulnerable workers but also to reduce strain on the national healthcare system and mitigate broader socioeconomic costs.
